# Federated Learning over MU-MIMO Vehicular Networks

**DOI:** 10.3390/e27090941

**Published:** 2025-09-09

**Authors:** Maria Raftopoulou, José Mairton B. da Silva, Remco Litjens, H. Vincent Poor, Piet Van Mieghem

**Affiliations:** 1 Faculty of Electrical Engineering, Mathematics and Computer Science, Delft University of Technology, 2628 CD Delft, The Netherlands; maria.raftopoulou@tno.nl (M.R.); p.f.a.vanmieghem@tudelft.nl (P.V.M.); 2 Department of Networks, Netherlands Organisation for Applied Scientific Research (TNO), 2595 DA The Hague, The Netherlands; 3 Department of Information Technology, Uppsala University, 751 05 Uppsala, Sweden; mairton.barros@it.uu.se; 4 Department of Electrical and Computer Engineering, Princeton University, Princeton, NJ 08544, USA; poor@princeton.edu

**Keywords:** vehicle selection, resource allocation, MU-MIMO, federated learning, wireless networks, vehicular networks

## Abstract

Many algorithms related to vehicular applications, such as enhanced perception of the environment, benefit from frequent updates and the use of data from multiple vehicles. Federated learning is a promising method to improve the accuracy of algorithms in the context of vehicular networks. However, limited communication bandwidth, varying wireless channel quality, and potential latency requirements may impact the number of vehicles selected for training per communication round and their assigned radio resources. In this work, we characterize the vehicles participating in federated learning based on their importance to the learning process and their use of wireless resources. We then address the joint vehicle selection and resource allocation problem, considering multi-cell networks with multi-user multiple-input multiple-output (MU-MIMO)-capable base stations and vehicles. We propose a “vehicle-beam-iterative” algorithm to approximate the solution to the resulting optimization problem. We then evaluate its performance through extensive simulations, using realistic road and mobility models, for the task of object classification of European traffic signs. Our results indicate that MU-MIMO improves the convergence time of the global model. Moreover, the application-specific accuracy targets are reached faster in scenarios where the vehicles have the same training data set sizes than in scenarios where the data set sizes differ.

## 1. Introduction

In recent years, many advances have been made in the field of autonomous vehicles. Autonomous vehicles rely on the information they receive from sensors, as well as from other vehicles and the network, through vehicle-to-vehicle and vehicle-to-infrastructure wireless links, respectively, to make real-time decisions, such as route planning, speed adjustment, and collision avoidance [1]. To ensure driving safety, driving decisions should be accurate, and communication via vehicle-to-vehicle and vehicle-to-infrastructure links should be fast and reliable. To address these challenges, machine learning (ML) algorithms are widely applied. Examples of such ML applications include the optimal assignment of radio resources and optimal handover control [1].

When driving, the environment is very dynamic and can change drastically over time. In addition, driving must be adjusted according to the location/area and the enforced driving rules. Hence, the applied ML algorithms should be constantly updated based on new sensor data and can greatly benefit from using data from other vehicles. For example, vehicles can share their camera data to enhance environmental perception, thus allowing vehicles to observe obstacles or dangerous situations that are out of the reach of their own cameras but still in close proximity [1].

Due to the distributed nature of the data, federated learning (FL) is a promising method for collaborative learning in vehicular networks [2]. Specifically, FL allows the training of a centralized global model using decentralized data samples from distinct vehicles without the need to upload the individual samples to a centralized server. A range of applications has been discussed in the literature that can benefit from FL in vehicular networks, with one example being co-operative environmental perception [3,4]. In particular, the FL global model can provide higher accuracy in detecting and localizing obstacles or hazardous situations compared to a local model trained on a single vehicle. This is because knowledge from multiple vehicles driving in the same area contributes to the global model and hence provides information that other vehicles do not have. Another example is the improvement of navigation systems, because vehicles from different areas, which experience different driving conditions, contribute to the global model [3]. More examples include traffic prediction for resource management [3] and steering wheel angle precision [5].

Some major challenges with FL in wireless networks are the high communication cost for exchanging the model parameters between the FL server and the vehicles, the wireless channel quality variations, and the limited transmission resources. To overcome these challenges, a subset of vehicles is selected to take part in each training round of the learning process. In [6], we addressed the agent (and in this context, vehicle) selection problem for FL in resource-constrained wireless networks by providing an agent selection framework based on distinct agent characteristics while also considering an application-specific latency budget. In this work, we extend the previously proposed framework to address the challenge of agent selection and resource management in the context of vehicular wireless networks. Given this context, we consider road and mobility models and a multi-cell network with multi-user multiple input multiple output (MU-MIMO)-capable base stations.

The integrated agent selection and resource management problem for wireless networks has been addressed in the literature, but mainly for stationary agents and in contexts other than vehicular networks [7]. For example, Chen et al. [8] address the minimization of training loss while considering parameters related to the wireless channels, whereas Zeng et al. [9] focus on minimizing energy consumption. Shi et al. [10] focus on latency-constrained networks and propose a policy to optimize the global model accuracy under a given latency constraint. Fan et al. [11] claim to have published the first work that addresses the minimization of the time duration of each communication round while also considering a practical mobility model. However, their evaluation does not consider a learning task related to vehicular networks or MU-MIMO-capable base stations. Moreover, the mobility model used is simpler and less realistic than the 3GPP-based model used in this study. One of the few works that addresses FL in vehicular networks is by Deveaux et al. [12]. Specifically, they highlight the need for algorithms addressing unevenly distributed data and propose a high-level protocol that allows the network to retrieve information about the type of data within each vehicle. The prior art on applying FL for beam assignment in MU-MIMO systems is very limited. A work addressing MU-MIMO systems is by Guan et al. [13], who propose an access scheduling algorithm. Unlike this work, they consider full-duplex transmissions and focus on Internet of Things applications.

The main contributions of this study are as follows:

- We evaluate the performance of FL over vehicular scenarios, which are realistically modeled using the road and mobility models from 3GPP [14]. These models are more complex and realistic than mobility models typically used in the literature. Additionally, we consider MU-MIMO-capable base stations, which are not frequently considered in FL-related studies. Moreover, we consider the learning task of object classification on the European traffic sign data set, which is a relevant data set for vehicular applications. This data set is statistically and geographically more diverse, and therefore more challenging to train on, than commonly used data sets such as MNIST and CIFAR-10.
- Based on the defined MU-MIMO vehicular scenario, we investigate the challenge of vehicle selection and resource management by characterizing vehicles based on their importance in the learning process and their wireless channel quality. We then propose the “vehicle-beam-iterative” (VBI) algorithm to approximate the solution of the defined optimization problem. The evaluation of the VBI algorithm provides insights into the novel and realistic scenario under investigation.
- We show that MU-MIMO-capable base stations improve the convergence time of the global model by enabling the selection of multiple vehicles on the same time–frequency resources and improving the achievable vehicle data rates.
- We show that the local loss is an effective vehicle selection metric for scenarios with non-independent and identically distributed (IID) data, assuming that all vehicles have the same training times. When vehicles have different training times, e.g., due to different data set sizes and/or processing capabilities, the loss-based policies do not provide substantial gains.
- We demonstrate, through realistic numerical evaluations, that convergence time in scenarios where vehicles have different data set sizes is longer than in scenarios where vehicles have the same data set sizes.

The outline of the paper is as follows. Section 2 describes the network, learning, and communication models. Section 3 then derives the joint vehicle selection and resource allocation optimization problem, and Section 4 describes the VBI algorithm. Section 5 presents the configuration of the considered evaluation scenarios, and Section 6 provides the numerical evaluation of the VBI algorithm. Finally, conclusions and proposals for future works are given in Section 7.

## 2. System Model

This section describes the considered network and learning models, as well as the communication model between vehicles and base stations. For clarity, a short description of the most commonly used symbols in this paper is given in Table 1.

### 2.1. Network Model

Consider a cellular network with one FL server, a set M of base stations and a set V of vehicles, where M=|M| and V=|V| are the number of base stations and vehicles in the network, respectively. The vehicles and the FL server collaboratively train a global model, without requiring the transmission of the data sets gathered by the vehicles, while the base stations facilitate communication between the vehicles and the FL server. For this, we assume that the FL server is connected to all base stations with fiber, hence their backhaul communication latency is negligibly small and that communication between the FL server and the base stations is synchronized.

**Figure 1:**
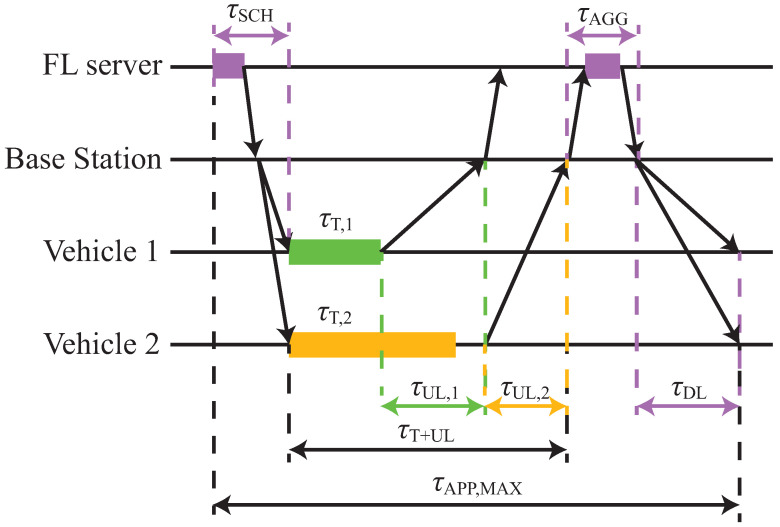
Schematic overview of the different steps involved in a single communication round and the corresponding time intervals.

Figure 1 shows a schematic overview of a communication round *i*, assuming a simple network with V=2 vehicles and M=1 base station. First, the FL server selects and notifies, via the base station, the vehicles that will participate in the learning. The vehicle selection and notification, potentially via broadcast transmission, are performed within a time interval of duration $\tau_{\mathrm{SCH}}$. Each selected vehicle $v\in V_{G}\left[i\right]$ then trains its local model, where $V_{G}\left[i\right]$ is the set of vehicles selected in communication round *i*. A vehicle $v\in V_{G}\left[i\right]$ has a training time $\tau_{T,v}$, which can be different from other vehicles, as shown in Figure 1, due to different data set sizes and/or processing capabilities.

Once each selected vehicle $v\in V_{G}\left[i\right]$ finishes its local training, it transmits its local model using the assigned uplink transmission resources to its serving base station, which then forwards the model to the FL server. The uplink transmission time $\tau_{\mathrm{UL},v}$ for vehicle *v* depends on its wireless channel quality, discussed in Section 2.3. In Figure 1, the transmissions of the two vehicles are assumed to be time-multiplexed, hence vehicle 2 does not initiate its uplink transmission until the completion of the uplink transmission of vehicle 1. Once all local models are aggregated at the FL server, the global model is updated for the next communication round i+1. The time duration of this process is $\tau_{\mathrm{AGG}}$. Finally, the FL server broadcast in the downlink, with duration $\tau_{\mathrm{DL}}$, the new global model to each vehicle v∈V. The process repeats until sufficient accuracy is achieved for the global model, verified using an FL server-specific testing data set.

An application-specific deadline budget $\tau_{\mathrm{APP},\mathrm{MAX}}$ can be set on the time duration of each communication round *i* to prevent the selection of vehicles with limited processing power and/or poor wireless channel quality, thus

(1) $$\tau_{\mathrm{SCH}}+\tau_{T+\mathrm{UL}}+\tau_{\mathrm{AGG}}+\tau_{\mathrm{DL}}\leq \tau_{\mathrm{APP},\mathrm{MAX}},$$

where $\tau_{T+\mathrm{UL}}$ is the time needed for all selected vehicles to perform local training and upload their local models to the FL server, as shown in Figure 1. We assume that the processing times at the FL server are negligible because it is likely to have significantly more powerful hardware compared to the vehicles. Moreover, we assume that the time duration of the broadcast to notify the selected vehicles for training is negligible because the control data transmitted is very small in size. Therefore, $\tau_{\mathrm{SCH}}\approx \tau_{\mathrm{AGG}}\approx 0$. Additionally, we assume that the network is configured to ensure a minimum bit rate at the cell edge and thus, the broadcast time $\tau_{\mathrm{DL}}$ is fixed for every communication round *i*. Therefore, using (1), we perform vehicle selection and resource allocation over a time period fulfilling the inequality:

(2) $$\tau_{T+\mathrm{UL}}\leq \tau_{\mathrm{APP},\mathrm{MAX}}-\tau_{\mathrm{DL}}.$$

Moreover, the FL process can be bound to the available transmission resources $C_{R,\mathrm{MAX}}$ allocated to the FL task, e.g., in a slice in 5G networks, which can restrict the number of selected agents per communication round.

In practical wireless communication scenarios, multiple scheduling time slots exist within the time interval $\tau_{T+\mathrm{UL}}$, occurring on a millisecond scale. These scheduling decisions depend on the experienced signal-to-noise ratio (SNR) of the vehicles, which varies within milliseconds. In this work, we focus on the resource allocation problem from a higher time-scale perspective. We perform periodic resource allocation over a period $\tau_{T+\mathrm{UL}}$ and we assume that the effects occurring on the millisecond scale, e.g., multipath fading, can be averaged.

### 2.2. Learning Model

In the considered learning model, vehicle v∈V has training data set $K_{v}$ and testing data set $K_{T,v}$, with $K_{v}=\left|K_{v}\right|$ and $K_{T,v}=\left|K_{T,v}\right|$ denoting the respective number of samples in each data set. Vehicle *v*’s input training data samples are given by $X_{v}=\left[x_{v1},\cdots ,x_{vK_{v}}\right]$, with $x_{vk}\in R^{n_{X}}$ as the $k^{th}$ input vector to its model and $n_{X}$ as the length of the input vector. Additionally, the output data samples are given by $Y_{v}=\left[y_{v1},\cdots ,y_{vK_{v}}\right]$, with $y_{vk}\in {\left\{0,1\right\}}^{n_{C}}$ as the real output vector corresponding to the $k^{th}$ input vector $x_{vk}$, and $n_{C}$ as the number of model outputs.

In local training conducted by vehicle *v*, predictions (model output) ${\hat{Y}}_{v}=\left[{\hat{y}}_{v1},\cdots ,{\hat{y}}_{vK_{v}}\right]$ are generated using the vehicle’s available training data samples in $K_{v}$, with ${\hat{y}}_{vk}\in R^{n_{C}}$ denoting the predicted output vector associated with input vector $x_{vk}$. The obtained local model is characterized by the derived parameter weights $W_{v}$, which are used such that, given the input data $X_{v}$, the predictions ${\hat{Y}}_{v}$ represent the real output $Y_{v}$. The closeness between the predictions ${\hat{Y}}_{v}$ and the real output $Y_{v}$ is generally expressed by the loss function $F(W_{v};X_{v},Y_{v})$, which depends on the input $X_{v}$ and the real output $Y_{v}$. For notational simplicity, we will omit this dependency in the remainder of the text and simply denote the loss function by $F(W_{v})$.

The objective of training the local model at vehicle *v* is

(3) $$\min_{W_{v}}F\left(W_{v}\right)=\frac{1}{K_{v}}\sum_{k\in K_{v}}f_{k}\left(W_{v}\right),$$

where $f_{k}\left(W_{v}\right)$ denotes the loss function of sample *k*, which for image classification problems is commonly defined as the cross-entropy loss [15]. To obtain the weights $W_{v}$ that minimize the loss function $F(W_{v})$, a number of iterations (local epochs) $n_{\mathrm{LE}}$ are performed. Assuming the stochastic gradient descent (SGD) optimizer [16], the weights $W_{v}$ are adapted at every local epoch based on the applied learning rate η.

In FL, the training data set $K=\cup_{v\in V}K_{v}$ (with K=|K|) is the union of the vehicle-specific training data sets and the global objective function $F(W_{G}\left[i\right])$ at communication round *i* is approximated by the weighted average of losses for the vehicle-specific local models:

(4) $$F\left(W_{G}\left[i\right]\right)\approx \sum_{v\in V_{G}\left[i\right]}\frac{K_{v}}{K}F\left(W_{v}\left[i\right]\right).$$

Given the SGD optimizer, the FedAvg method [17] determines the global model weights $W_{G}\left[i\right]$ at the end of communication round *i* as the weighted average of the local model weights:

(5) $$W_{G}\left[i\right]\leftarrow \sum_{v\in V_{G}\left[i\right]}\frac{K_{v}}{K}W_{v}\left[i\right],$$

The updated global weights are then broadcast to all the vehicles for the next communication round.

### 2.3. Communication Model

We assume that both base stations and vehicles are equipped with beamforming antenna arrays to form narrow and strong beams. Specifically, beam pairs are formed between the base stations and vehicles, and the same beam pair is used for both uplink and downlink transmissions [18]. Moreover, we assume that the base station and vehicle beams are directly pointing at each other and interference between different transmissions is neglected. For the base station antenna array, we assume use of a grid-of-beams mode, i.e., a base station m∈M can form a pre-defined set of beams $B_{m}$ in the three-dimensional space. We further assume that all base stations have the same set of beams and thus each base station *m* has $B_{M}=\left|B_{m}\right|$ beams. Regarding the antenna array of the vehicles, we assume a single beam that can be steered in any direction. A detailed description of the antenna array models is provided in Appendix A.1.

We assume an OFDMA-based (orthogonal frequency division multiple access) access technology and MU-MIMO-capable base stations, thus, spatial-multiplexing, i.e., multiple vehicles can transmit at a serving cell on the same time-frequency resources. Moreover, we consider time-multiplexing, thus, beams from different vehicles can be paired to the same base station beam. Additionally, wideband transmissions are assumed. Therefore, during communication round *i*, a vehicle $v\in V_{G}\left[i\right]$ is assigned to beam $b\in B_{\mathrm{TOT}}$ (from a base station m∈M) for a fraction of the time period $\tau_{T+\mathrm{UL}}$, where $B_{\mathrm{TOT}}=\cup_{m\in M}B_{m}$ is the set with all base station beams and $B_{\mathrm{TOT}}=\left|B_{\mathrm{TOT}}\right|=B_{M}M$ is the total number of beams in the network. Finally, we assume that during the period $\tau_{T+\mathrm{UL}}$, vehicles stay connected to the same beam. This assumption is further discussed in Section 5.4.

For the uplink transmission of the local model, and as an input to the periodic resource assignment, we estimate the bit rate $R_{vb}$ of vehicle *v* from beam $b\in B_{\mathrm{TOT}}$ as

(6) $$R_{vb}=f_{\mathrm{BW}}\min\left(\log_{2}\left(1+10^{\Gamma_{vb}/10}\right),15\right),$$

where 15 bits/Hz/s is the target peak spectral efficiency in the uplink channel in 5G [19], $f_{\mathrm{BW}}$ denotes the system bandwidth in MHz, and $\Gamma_{vb}$ is the estimated uplink SNR at vehicle *v* from beam *b* given, in dB, by

(7) $$\Gamma_{vb}=P_{V,\mathrm{MAX}}+G_{T,vb}+G_{V,vb}+G_{M,vb}-P_{\mathrm{NOISE}}-P_{\mathrm{NF},M},$$

where $P_{V,\mathrm{MAX}}$ is the maximum transmit power of the vehicle in dBm, $G_{T,vb}$ is the transmission gain between vehicle *v* and the base station that beam *b* belongs to in dB, $G_{V,vb}$ and $G_{M,vb}$ are the vehicle and base station antenna gains in dBi, respectively, $P_{\mathrm{NOISE}}$ is the thermal noise power in dBm, and $P_{\mathrm{NF},M}$ is the noise figure in dB at each base station. The channel gain $G_{T,vb}$ is modeled by [20]

(8) $$G_{T,vb}=20\log\left(\frac{c}{4\pi f_{C}}\right)-10\gamma \log\left(d_{vb}\right)+\psi ,$$

(in dB) with *c* the speed of light (in m/s), $f_{C}$ the carrier frequency (in Hz), $d_{vb}$ the 3D distance between vehicle *v* and its serving base station (in m), γ the path loss exponent, and ψ as a zero-mean Gaussian random variable with standard deviation σ, included to model shadow fading. Due to the periodic nature of the resource assignment approach and the assumption that vehicles stay connected to the same beam during the period $\tau_{T+\mathrm{UL}}$, the SNR $\Gamma_{vb}$ and bit rate $R_{vb}$ are assumed to be constant during the period $\tau_{T+\mathrm{UL}}$.

Finally, for the broadcast transmission, we assume that all base stations use all their beams because there are many vehicles in the network, spread in different directions. We derive the broadcast bit rate based on the network layout and the antenna array configuration, in Section 5.4.

## 3. Problem Formulation

In real-world applications, vehicles are diverse in terms of their training data, processing capabilities to train their local model, and wireless channel quality. In this section, we define the so-called *vehicle importance* metric to characterize the vehicles. We present the latency considerations, which depend on the processing capabilities and the wireless channel quality of the vehicles. Furthermore, we combine the vehicle importance with the latency considerations to formulate the joint vehicle selection and resource allocation optimization problem.

For the joint vehicle selection and resource allocation problem, we consider two optimization parameters; one for the vehicle selection and one for the resource allocation. Specifically, we define the optimization vector $s\left[i\right]\in {\left\{0,1\right\}}^{V}$, during communication round *i*, in which $s_{v}\left[i\right]=1$ when vehicle *v* is selected for training, and $s_{v}\left[i\right]=0$ otherwise. Additionally, we define the optimization matrix $A\left[i\right]\in {\left\{0,1\right\}}^{V\times B_{\mathrm{TOT}}}$, in which $A_{vb}\left[i\right]\in \left\{0,1\right\}$, holds the beam associations between the selected vehicles and the base station beams. Because all selected vehicles remain connected to a single beam during the time interval $\tau_{T+\mathrm{UL}}$, the following equality must hold

(9) $$A\left[i\right]\cdot 1_{B_{\mathrm{TOT}}\times 1}=s\left[i\right],$$

where $1_{B_{\mathrm{TOT}}\times 1}$ denotes the all ones $B_{\mathrm{TOT}}\times 1$ vector.

### 3.1. Vehicle Importance

To capture the diversity of vehicles, we introduce the *vehicle importance* $q_{vb}\left[i\right]\in R$, which is the metric governing the vehicle selection and resource allocation process at communication round *i*. Specifically, the vehicle importance $q_{vb}\left[i\right]$ captures the trade-off between the importance of vehicle *v* in the learning process against its consumed transmission resources on the, potentially assigned, beam *b*. The definition of this metric is essential to the vehicle selection and resource allocation problem, as both learning and wireless aspects play a significant role in the accuracy and convergence time of the global model. For example, if only learning aspects are considered, vehicles with poor channels may be selected for training, which will lead to long upload times and eventually a long global model convergence time. However, if a vehicle with a poor channel holds a data set belonging to a class with few samples across the system, i.e., a non-IID data scenario with varying sample counts across agents, it is important to include that vehicle in the FL training despite its wireless conditions. The vehicle importance $q_{vb}\left[i\right]$ metric also allows for configuring the relative significance of the learning and wireless aspects, as shown below.

We express the importance of vehicle *v* in the learning process [6] by the locally computed *loss* function $F(W_{G,v}\left[i\right])$, determined based on the testing data set $K_{T,v}$ and global weights $W_{G}\left[i\right]$, as generated and broadcast at the end of communication round *i*. The computed loss $F(W_{G,v}\left[i\right])$ is conveyed to the central FL server and used in the vehicle selection and resource allocation process for upcoming communication round i+1. We disregard the corresponding transmission time, considering that the loss is just a scalar value.

The time-frequency resources $C_{R,vb}\left[i\right]$ consumed by vehicle *v* for the upload of the locally derived model weights $W_{v}\left[i\right]$ on candidate beam *b* in communication round *i*. We then consider $C_{R,vb}\left[i\right]$ as:

(10) $$C_{R,vb}\left[i\right]=\tau_{\mathrm{UL},vb}\left[i\right]f_{\mathrm{BW}}=\frac{Z}{R_{vb}\left[i\right]}f_{\mathrm{BW}},$$

where $\tau_{\mathrm{UL},vb}\left[i\right]$ is the transmission time for vehicle *v* on beam *b* in seconds, $R_{vb}\left[i\right]$ is the bit rate given by (6), and *Z* is the model size in Mbits. Calculation of the resource consumption $C_{R,bv}\left[i\right]$ does not require additional vehicle-to-base station communication since the bit rates can be estimated based on the periodic channel quality indicator (CQI) feedback that all vehicles report to their serving base station.

We define the importance $q_{vb}\left[i\right]$ of vehicle *v* on beam *b* at communication round *i* as

(11) $$q_{vb}\left[i\right]=\frac{F{\left(W_{G,v}\left[i\right]\right)}^{\rho }}{C_{R,vb}^{1-\rho }\left[i\right]},$$

where ρ∈[0,1] is a constant that can be configured to set the relative significance of the learning importance and the resource consumption. We further define the matrix $Q\left[i\right]=\left[q_{1}\left[i\right],\cdots ,q_{B_{\mathrm{TOT}}}\left[i\right]\right]\in R^{V\times B_{\mathrm{TOT}}}$, where $q_{b}\left[i\right]$ is a column vector holding the importance $q_{vb}\left[i\right]$ of each vehicle *v* on beam $b\in B_{\mathrm{TOT}}$.

### 3.2. Latency Considerations

We assume that a fixed amount of uplink transmission resources $C_{R,\mathrm{MAX}}$ are allocated to the FL task, which are expressed as a product of the bandwidth and the maximum allowed aggregate upload time. Therefore, the selected vehicles should perform their uplink transmission within the available transmission resources $C_{R,\mathrm{MAX}}$, where the uplink transmission resources $C_{R,vb}\left[i\right]$ of vehicle *v* on beam *b* at communication round *i* are given by (10). Because we assume wideband transmissions, for simplicity, we will express from here onward the transmission resources $C_{R,\mathrm{MAX}}$ and $C_{R,vb}\left[i\right]$ only in terms of time. Additionally, the selected vehicles should train and transmit their local models within the latency budget $\tau_{\mathrm{APP},\mathrm{MAX}}-\tau_{\mathrm{DL}}$, as previously captured in (2). In this section, we first derive the training time $\tau_{T,v}$ of vehicle *v*, and then elaborate on the two latency constraints.

The training time $\tau_{T,v}$ of vehicle *v* depends on the vehicle’s processing capability $g_{v}$, as well as on its data set size $K_{v}$, and other training-related parameters, e.g., number of local epochs $n_{\mathrm{LE}}$. The processing capability $g_{v}$ of vehicle *v* is measured in floating point operations (FLOPs) per second as [21]

(12) $$g_{v}=n_{\mathrm{CORES},v}\;\nu_{v}\;\omega_{v},$$

where $n_{\mathrm{CORES},v}$ is the number of central processing unit (CPU) cores at vehicle *v*, $\nu_{v}$ is the CPU clock frequency at vehicle *v* in cycles per second and $\omega_{v}$ is the number of FLOPs per cycle at vehicle *v*. Then, the training time $\tau_{T,v}$ of vehicle *v* is

(13) $$\tau_{T,v}=\left\lceil \frac{K_{v}}{s_{B}}\right\rceil \frac{n_{\mathrm{FLOP},G}\;n_{\mathrm{LE}}}{g_{v}},$$

where $n_{\mathrm{FLOP},G}$ denotes the number of FLOPs to train the model for a batch of size $s_{B}$ and ⌈·⌉ represents the ceiling operation.

The first latency constraint relates to the available transmission resources $C_{R,\mathrm{MAX}}=\tau_{T+\mathrm{UL}}=\tau_{\mathrm{APP},\mathrm{MAX}}-\tau_{\mathrm{DL}}$ that are available per communication round *i*. Considering spatial- and time-multiplexing, multiple vehicles can be scheduled on a given base station beam and share the frequency resources over time. To perform this co-scheduling of vehicles on the same beam, the training time $\tau_{T,v}$, as defined in (13), of each vehicle should be taken into consideration. That is because each base station beam becomes active, i.e., receives uplink data, for the first time when the vehicle with the shortest training time that is assigned to that beam finishes its local training. We define the vector $\tau_{L}\left[i\right]={\left[\tau_{L,1[i]},\cdots ,\tau_{L,B_{\mathrm{TOT}}}\left[i\right]\right]}^{⊺}\in R^{B_{\mathrm{TOT}}}$ to indicate the start time of the uplink transmissions per beam at communication round *i*, where ^⊺^ denotes the transpose operation, and

(14) $$\tau_{L,b}\left[i\right]=\min_{1\leq vs.\leq V}{\hat{\tau }}_{L,b}\left[i\right],$$

where ${\hat{\tau }}_{L,b}\left[i\right]\in R^{V}$ indicates the training times of the vehicles assigned to beam *b*. The vector ${\hat{\tau }}_{L,b}\left[i\right]$ is defined from the auxiliary matrix ${\hat{T}}_{L}=\left[{\hat{\tau }}_{L,1},\cdots ,{\hat{\tau }}_{L,B_{\mathrm{TOT}}}\right]\in R^{V\times B_{\mathrm{TOT}}}$ which associates the training time $\tau_{T,v}$ of each vehicle *v* to its assigned beam as follows:

(15) $${\hat{T}}_{L}\left[i\right]=\left(\tau_{T}\cdot 1_{1\times B_{\mathrm{TOT}}}\right)\circ A\left[i\right],$$

where $\tau_{T}={\left[\tau_{T,1},\cdots ,\tau_{T,V}\right]}^{⊺}\in R^{V}$ and ∘ is the Hadamard product. Given that uplink transmissions are starting at a different time per beam, vehicles can be co-scheduled on the same beam if they can jointly finish their uplink transmissions within the remaining transmission resources. Hence, the condition for co-scheduling is

(16) $$1_{1\times V}\cdot \left(T_{\mathrm{UL}}\left[i\right]\circ A\left[i\right]\right)≼\left(\tau_{\mathrm{APP},\mathrm{MAX}}-\tau_{\mathrm{DL}}\right)\;1_{1\times B_{\mathrm{TOT}}}-\tau_{L}^{⊺}\left(A\left[i\right]\right),$$

where $T_{\mathrm{UL}}\left[i\right]=\left[\tau_{\mathrm{UL},1}\left[i\right],\cdots ,\tau_{\mathrm{UL},B_{\mathrm{TOT}}}\left[i\right]\right]\in R^{V\times B_{\mathrm{TOT}}}$ with $\tau_{\mathrm{UL},b}\left[i\right]\in R^{V}$ holding the upload time duration $\tau_{\mathrm{UL},vb}$ of each vehicle *v* on beam *b*, ≼ denotes the element-wise inequality and $\tau_{L}\left(A\left[i\right]\right)$ denotes the dependence on A[i].

**Figure 2:**
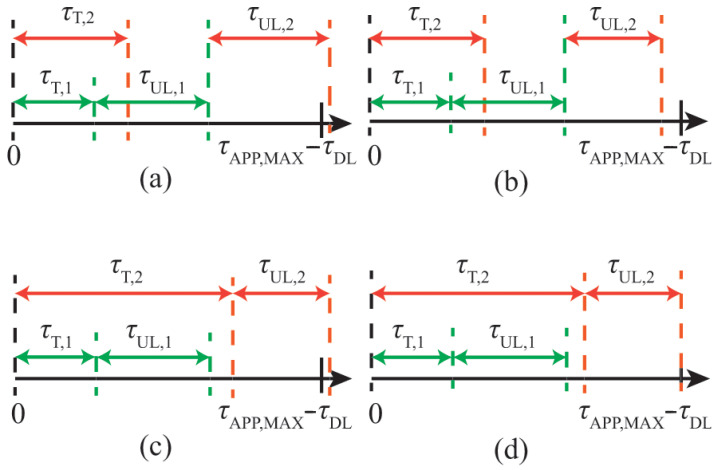
(**a**) The two vehicles cannot be co-scheduled on the same beam because the constraint in (16) is violated; (**b**) The two vehicles can be co-scheduled on the same beam because the constraint in (16) is fulfilled; (**c**) The two vehicles cannot be co-scheduled on the same beam because the constraint in (17) is violated; (**d**) The two vehicles can be co-scheduled on the same beam because the constraint in (17) is fulfilled. In all four sub-figures, the color refers to a different vehicles.

Figure 2a,b show two examples of assigning two vehicles to the same beam. In both examples, the start time of the uplink transmissions is equal to $\tau_{L,b}=\min\left\{\tau_{T,1},\tau_{T,2}\right\}=\tau_{T,1}$. Thus, the joint upload time interval is $\tau_{\mathrm{APP},\mathrm{MAX}}-\tau_{\mathrm{DL}}-\tau_{T,1}$. Figure 2a illustrates that the two vehicles cannot be co-scheduled on the same beam because $\tau_{\mathrm{UL},1}+\tau_{\mathrm{UL},2}>\tau_{\mathrm{APP},\mathrm{MAX}}-\tau_{\mathrm{DL}}-\tau_{T,1}$, which violates the constraint in (16). Conversely, Figure 2b shows that co-scheduling is possible and that the constraint in (16) is fulfilled.

The fulfillment of (16) alone does not guarantee that vehicles can train and transmit within the time interval $\tau_{\mathrm{APP},\mathrm{MAX}}-\tau_{\mathrm{DL}}$, which was captured in (2). Therefore, a second latency constraint is needed, as follows:

(17) $$\tau_{T}+\left(T_{\mathrm{UL}}\left[i\right]\circ A\left[i\right]\right)\cdot 1_{B_{\mathrm{TOT}}\times 1}≼\left(\tau_{\mathrm{APP},\mathrm{MAX}}-\tau_{\mathrm{DL}}\right)1_{B_{\mathrm{TOT}}\times 1}.$$

Consider again the example of assigning two vehicles to one beam and that $\tau_{L,b}=\min\left\{\tau_{T,1},\tau_{T,2}\right\}=\tau_{T,1}$, as shown in Figure 2c,d. Even though (16) is fulfilled, the example in Figure 2c illustrates that vehicle 2 should not be scheduled on that particular beam, because its training and upload time is $\tau_{T,2}+\tau_{\mathrm{UL},2}$, which exceeds the time interval $\tau_{\mathrm{APP},\mathrm{MAX}}-\tau_{\mathrm{DL}}$ and violates the constraint in (17). On the contrary, the constraint in (17) is fulfilled in the example in Figure 2d.

### 3.3. Problem Formulation

For a given communication round *i*, we formulate the following joint vehicle selection and resource allocation optimization problem to maximize the total vehicle importance:

(18)maxs[i],A[i]Tr(Q[i]·A⊺[i])(19)subjecttoA[i]·1BTOT×1=s[i],(20)11×V·(TUL[i]∘A[i])≼(τAPP,MAX−τDL)11×BTOT−τL⊺(A[i]),(21)τT+(TUL[i]∘A[i])·1B×1≼(τAPP,MAX−τDL)1BTOT×1,(22)s[i]∈{0,1}V×1,(23)A[i]∈{0,1}V×BTOT,

where Tr(·) denotes the trace of a matrix, i.e., the sum of the elements on the diagonal. The binary optimization variable s[i] indicates whether a vehicle is selected and the binary optimization matrix A[i] indicates the beam on which the selected vehicles are assigned to. Therefore, the total vehicle importance is defined as the summation of distinct vehicle importance values from a set of vehicles, that would potentially jointly be selected for training. Thus, the optimization problem aims to select the set of vehicles that provides the maximum total vehicle importance over other vehicle sets, subject to constraints (19)–(23). With objective function (18), the given optimization problem takes both learning and wireless aspects into account, and its solution directly affects the accuracy and convergence time of the global model. Constraint (19) indicates that vehicles participating in the learning process must be associated with at most one beam in one cell. Constraint (20) shows that vehicles can be assigned to, and time-multiplexed on, the same beam only if both can finish their uplink transmissions within the related time interval. Finally, constraint (21) indicates that all selected vehicles need to train and transmit their local models within the time interval $\tau_{\mathrm{APP},\mathrm{MAX}}-\tau_{\mathrm{DL}}$.

In the optimization problem in (18)–(23), the selection of vehicles s[i] is defined based on the beam associations A[i] using (19). Therefore, we can reduce the optimization parameters by combining (19) and (22), leading to:

(24)maxA[i]Tr(Q[i]·A⊺[i])(25)subjecttoA[i]·1BTOT×1≼1V×1,(26)11×V·(TUL[i]∘A[i])≼(τAPP,MAX−τDL)11×BTOT−τL⊺(A[i]),(27)τT+(TUL[i]∘A[i])·1BTOT×1≼(τAPP,MAX−τDL)1V×1,(28)A[i]∈{0,1}V×BTOT.

The optimization problem in (24)–(28) is non-linear because the vector $\tau_{L}^{⊺}\left(A\left[i\right]\right)$ requires the evaluation of a min(·) function, as shown in (14). Typically, non-linear integer optimization problems are more difficult to solve than linear integer problems, even if the solution space is relatively small. That is because they are often non-convex and reaching a global optimum cannot be guaranteed. To eliminate the nonlinearity, we assume the same training time $\tau_{T}$ for all vehicles and define a relaxed version of the optimization problem in (24)–(28). Such a relaxation is achieved by assuming that all vehicles have the same data set size $K_{v}$ and processing capabilities $g_{v}$. Additionally, constraint (27) is not needed in the relaxed problem because it is implied by constraint (26). The relaxed optimization problem is then given by

(29)maxA[i]Tr(Q[i]·A⊺[i])(30)subjecttoA[i]·1BTOT×1≼1V×1,(31)11×V·(TUL[i]∘A[i])≼(τAPP,MAX−τDL−τL)11×BTOT,(32)A[i]∈{0,1}V×BTOT.

For problems with small numbers of variables and constraints, the solution to the relaxed optimization problem in (29)–(32) is derived using integer linear programming solvers. In this work, we consider the COIN-OR branch and cut (CBC) solver [22]. However, once the number of variables and constraints increases, e.g., in scenarios where a large number of vehicles are considered for the learning, the CBC solver either requires long runtimes or fails to converge to the optimal solution. In Section 4, we propose a heuristic algorithm, namely the VBI algorithm, to approximate the solution of both optimization problems (24)–(28) and (29)–(32). The advantage of a heuristic algorithm is that it provides a near-optimal solution, even in scenarios where the solver cannot converge to the optimal solution. Additionally, the algorithm provides its solution in a shorter time than the solver, especially in scenarios with large number of variables and constraints.

## 4. Proposed Solution

In this section, we propose the VBI algorithm as an approximated solution to the optimization problems (24)–(28) and (29)–(32). First, we provide a description of the algorithm and then, we address the impact of the vehicle importance $q_{vb}$ in (11) on the behavior of the VBI algorithm.

### 4.1. Algorithmic Description

The VBI algorithm indicates which vehicles are selected for training at each communication round and assigns the selected vehicles to an appropriate base station beam. Hence, the VBI algorithm forms vehicle–beam pairs with the goal of maximizing the total vehicle importance. The VBI algorithm is described in Algorithm 1 and its explanation is as follows.

First, in lines 1–4, the initialization is performed. In line 1, the vehicle-beam matrix A is initialized by setting $A_{vb}=0$ to all vehicle-beam pairs that do not satisfy constraint (27), i.e., $\tau_{T,v}+\tau_{\mathrm{UL},vb}\leq \tau_{\mathrm{APP},\mathrm{MAX}}-\tau_{\mathrm{DL}}$. The remaining entries of matrix A are temporarily set to the “−1” value and they will be later updated by the algorithm. In line 2, a vector $\tau_{B}$, holding the upload latency budget for every beam is defined and it is initialized at $\tau_{\mathrm{APP},\mathrm{MAX}}-\tau_{\mathrm{DL}}$. Finally, lines 3 and 4, initialize to zero the vector $\tau_{L}$ holding the start time of the uplink transmissions and the total vehicle importance $Q_{\mathrm{TOT}}$, respectively.
**Algorithm 1** Vehicle-Beam-Iterative (VBI) Algorithm**Input:** Training time $\tau_{T}$ of vehicles, upload time $T_{\mathrm{UL}}$ of vehicles per beam, importance Q of vehicles per beam and time period $\tau_{\mathrm{APP},\mathrm{MAX}}-\tau_{\mathrm{DL}}$**Output:** Vehicle selection and beam allocation A and total vehicle importance $Q_{\mathrm{TOT}}$    1:Set $A_{vb}=0$ if $\tau_{T,v}+\tau_{\mathrm{UL},vb}>\tau_{\mathrm{APP},\mathrm{MAX}}-\tau_{\mathrm{DL}}$, else $A_{vb}=-1$, for each vehicle v∈V and beam $b\in B_{\mathrm{TOT}}$    2:Set $\tau_{B,b}=\tau_{\mathrm{APP},\mathrm{MAX}}-\tau_{\mathrm{DL}}$, for each beam $b\in B_{\mathrm{TOT}}$    3:Set $\tau_{L,b}=0$, for each beam $b\in B_{\mathrm{TOT}}$    4:Set $Q_{\mathrm{TOT}}=0$    5:**while** matrix A contains an entry with value “−1” **do**    6:    **for** every vehicle v∈V not yet scheduled **do**    7:        Obtain $b^{*}={\mathrm{argmax}}_{b\in B_{\mathrm{TOT}}}\left(q_{vb}\right)$, given that $A_{vb}=-1$    8:    **end for**    9:    **for** every beam $b\in B_{\mathrm{TOT}}$ **do**  10:        Set scheduled = False  11:        Obtain $v^{*}={\mathrm{argmax}}_{v\in V_{b}}\left(q_{vb}\right)$, where $V_{b}$ holds the vehicles selecting beam *b* as their $b^{*}$  12:        **if** $v^{*}$ is the first vehicle scheduled on beam *b*, i.e., $\tau_{L,b}=0$ **then**  13:            Set training time $\tau_{L,b}=\tau_{T,v^{*}}$  14:            Set scheduled = True  15:        **else if** $\tau_{\mathrm{UL},v^{*}b}\leq \tau_{B,b}-\min\left(\tau_{L,b},\tau_{T,v^{*}}\right)$ **then**  16:            Set training time $\tau_{L,b}=\min\left(\tau_{L,b},\tau_{T,v^{*}}\right)$  17:            Set scheduled = True  18:        **end if**  19:        **if** scheduled = True **then**  20:             Set $A_{v^{*}b}=1$  21:             Set $A_{v^{*}\hat{b}}=0$ for all other beams $\hat{b}\in B_{\mathrm{TOT}}\setminus b$  22:             Update $\tau_{B,b}-=\tau_{\mathrm{UL},v^{*}b}$  23:             Update total importance $Q_{\mathrm{TOT}}+=q_{v^{*}b}$  24:        **end if**  25:    **end for**  26:    **for** every vehicle v∈V not yet scheduled **do**  27:        **for** every beam $b\in B_{\mathrm{TOT}}$ **do**  28:             **if** $\tau_{\mathrm{UL},vb}>\tau_{B,b}-\tau_{L,b}$ **then**  29:                 $A_{vb}=0$  30:             **end if**  31:        **end for**  32:    **end for**  33:**end while**  34:**return** A, $Q_{\mathrm{TOT}}$

After the initialization step, from line 5 onwards the algorithm repeats continuously until all entries of the matrix A are set to 0 or 1. The algorithm assigns at most one vehicle per beam per iteration and hence at most *V* iterations are performed. Each iteration consists of the following five steps:

- **STEP 1 (lines 6–8)**: For each vehicle v∈V that has not already been selected for training, the beam $b^{*}$ that maximizes the importance $q_{vb}$ of the vehicle *v* is obtained, assuming that $A_{vb}=-1$.
- **STEP 2 (lines 9–11)**: From line 9 onwards, the algorithm iterates over all beams to define per beam $b\in B_{\mathrm{TOT}}$ whether or not a vehicle will be assigned to it and which vehicle that will be. Depending on whether or not a vehicle is assigned to the beam, different steps are followed later on. Therefore, in line 10 a decision variable is initialized to False. Then, in line 11, based on the derived potential vehicle-beam pairs from step 1 (line 7), the vehicle $v^{*}$ that has the highest importance $q_{vb}$ on each beam *b* is selected.
- **STEP 3 (lines 12–18)**: In this step a decision is taken on whether or not the selected vehicle $v^{*}$ can be scheduled on beam *b*. In line 12, it is checked whether or not vehicle $v^{*}$ is the first vehicle to be scheduled on beam *b*. If it is the first one, line 13 sets the training time $\tau_{L,b}$ at beam *b* equal to the training time $\tau_{T,v^{*}}$ of vehicle $v^{*}$ and line 14 sets the decision variable to True. If vehicle $v^{*}$ is not the first vehicle to be scheduled on beam *b*, line 15 evaluates according to constraint (26) if vehicle $v^{*}$ can be co-scheduled with the other vehicle(s) already scheduled on beam *b*. If vehicle $v^{*}$ can be co-scheduled, in line 16, the training time $\tau_{L,b}$ at beam *b* is set to the minimum time between the training time $\tau_{L,b}$ set in a previous iteration, when scheduling a different vehicle, and the training time $\tau_{T,v^{*}}$ of the newly scheduled vehicle $v^{*}$. Line 17 sets the decision variable to True.
- **STEP 4 (lines 19–25)**: If vehicle $v^{*}$ is scheduled on beam *b*, i.e., the decision variable is True, in lines 20 and 21, the VBI algorithm updates accordingly the entries of matrix A involving vehicle $v^{*}$ to ensure that it is assigned only to beam *b*. Next, line 22 reduces the total available uploading latency budget $\tau_{B,b}$ accordingly and line 23 increases the total vehicle importance $Q_{\mathrm{TOT}}$ with the importance $q_{v^{*}b}$ of the newly scheduled vehicle. In case that vehicle $v^{*}$ is not scheduled, i.e., the decision variable is False, no action is taken and the vehicle can be re-considered for scheduling in a later iteration.
- **STEP 5 (lines 26–32)**: After iterating over all beams, and before starting a new iteration as a result of line 5, an update step takes place. Specifically, lines 26–32 discard vehicle–beam pairs that cannot fulfill constraint (27) due to the newly scheduled vehicles in the given algorithm iteration.

Finally, all iterations are completed in line 33 and then line 34 returns matrix A and the total vehicle importance $Q_{\mathrm{TOT}}$.

The complexity of the algorithm is split into two parts. The first part relates to the initialization steps, which have a complexity of $O(VB_{\mathrm{TOT}})$ due to the calculations in line 1. The second part relates to STEPS 1–5, which also have a complexity of $O(VB_{\mathrm{TOT}})$ for a single beam iteration. As mentioned above, the algorithm performs at most *V* iterations, and hence the complexity is $O(V^{2}B_{\mathrm{TOT}})$.

### 4.2. Algorithm Behavior

Because the VBI algorithm depends on vehicle importance $q_{vb}$ in (11), the value of the tuning parameter ρ influences its behavior. Specifically, when ρ=0, the importance $q_{vb}$ depends only on the resource consumption $C_{R,vb}$, which essentially depends on the bit rate $R_{vb}$ and varies per beam. To maximize the total vehicle importance $Q_{\mathrm{TOT}}$, the algorithm selects vehicles with the strongest wireless channels and assigns them to the beams that provide the highest bit rate $R_{vb}$. Hence, the VBI algorithm maximizes the number of selected vehicles. We will refer to the solution of the VBI algorithm with ρ=0 as VBI-rate.

On the other hand, when ρ=1, the vehicle importance $q_{vb}$ depends only on the training loss $F(W_{G,v})$, which is independent of the beam. In this case, the VBI algorithm prioritizes vehicles with high training loss $F(W_{G,v})$, thus meaning that they may be assigned on a sub-optimal beam in terms of their resource consumption $C_{R,vb}$. Nevertheless, latency constraints are still considered, and the shorter the latency budget $\tau_{\mathrm{APP},\mathrm{MAX}}$, the more likely a vehicle will be assigned to the beam with the lowest resource consumption $C_{R,vb}$ (and highest bit rate $R_{vb}$). If a vehicle is assigned to a sub-optimal beam in terms of the resource consumption $C_{R,vb}$, more resources will be consumed, thus limiting the total number of selected vehicles. We will refer to the solution of the VBI algorithm with ρ=1 as VBI-loss.

Notably, the VBI algorithm is general, as it allows redefining vehicle importance $q_{vb}$ in terms of either learning metrics (numerator) or wireless resource metrics (denominator) in Equation (11). In addition, it is flexible, since different values of ρ∈[0,1] yield variants of VBI that emphasize learning performance or wireless resource consumption to different degrees.

## 5. Scenario Configuration

This section presents the considered scenarios to evaluate the performance of the VBI algorithm. First, we present the learning task and then introduce the configuration of four learning scenarios. Next, we provide the baseline algorithms, which will be compared against the VBI algorithm and finally, we present the wireless scenario.

### 5.1. Learning Task

The learning task considered in this paper is the object classification of traffic signs. This learning task is a relevant FL application in vehicular networks because different countries use different traffic signs. Therefore, an algorithm that very accurately detects the meaning of a traffic sign in one country may not be able to detect the traffic sign that has the same meaning in a different country, or a traffic sign that does not exist in its origin country. With FL, a global model can be trained based on knowledge from both countries, which will allow for all vehicles to accurately detect traffic signs from both countries. For this work, the European traffic sign data set is used, comprising 164 classes of traffic signs originating from six distinct European countries [23]. Considering that some of the classes contain very few training samples, we select the $n_{C}=10$ classes with the highest number of available samples for our study.

**Figure 3:**
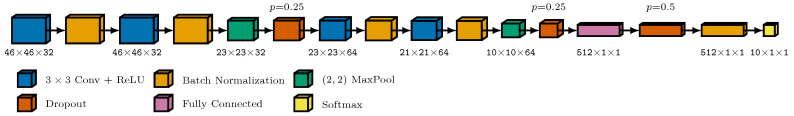
The CNN architecture assumed to perform the object classification task.

The object classification task utilizes a convolutional neural network (CNN) architecture similar to that used in research by Serna and Yuichek [23] and Chiamkurthy [24], which are in turn both inspired by the Visual Geometry Group (VGG) architecture [25]. Figure 3 depicts the assumed CNN architecture, which applies the rectified linear unit (ReLU) function, batch normalization, max pooling and dropout regularization. The final layer is activated by a softmax with 10 outputs indicating the per-class likelihoods. In total, the CNN comprises 3349418 trainable parameters. Assuming a 32-bit precision per trainable parameter, this translates to a model size of Z≈107 Mbits.

### 5.2. Learning Scenarios

In our analysis, we consider scenarios with V=50 vehicles and both IID and non-IID data. Moreover, we address both the scenarios where vehicles have the same and different training data set sizes, which are described by the problems in (29)–(32) and (24)–(28), respectively. In total, we consider four learning scenarios, whose configurations are summarized in Table 2. Apart from the scenario with same data set size and IID data, in the other three scenarios, the number of training samples are unevenly split over the number of assigned classes. For the scenario with the same data set size and non-IID data, this split is performed such that on average all classes are equally represented in the training data set K. For scenarios with different data set sizes, the number of samples per assigned class per vehicle is drawn from a Poisson distribution with a rate of 15 and 75, for IID data and non-IID data, respectively, as also shown in Table 2.

For calculation of the loss $F(W_{G,v}\left[i\right])$ of vehicle *v* at communication round *i*, the categorical cross-entropy loss function is applied on the testing data set $K_{T,v}$, which is unique for every vehicle and three times smaller than the training data set $K_{v}$. Moreover, the split of the testing data set among the vehicles and the classes is similar to the split of the training data set. Finally, the accuracy of the global model in all four scenarios is measured at the FL server based on an FL server specific testing data set, which consists of 100 samples per class. Note that the accuracy of the global model is defined as the proportion of correctly categorized testing samples divided by the total number of classification instances, where for each classification instance a single testing sample from the FL server is used.

For the training, the vehicles use the SGD optimizer with learning rate η=0.05, batch size $s_{B}=64$ and with each vehicle performing $n_{\mathrm{LE}}=2$ local epochs. The number of FLOPs required from the vehicles to train the CNN for a batch size $s_{B}=64$ is measured by the Keras library, in Python version 3.6.8, which is $n_{\mathrm{FLOP},G}=6.55$ GFLOPs. Regarding the hardware of the vehicles, we consider the processing capabilities $g_{v}=64$ GFLOPs per second. Therefore, the training time $\tau_{T,v}$ of vehicle *v*, as given by (13), depends on the number of training samples $K_{v}$ at vehicle *v*, which depends on the learning scenario.

### 5.3. Baseline Algorithms

To evaluate the performance of the VBI algorithm, we compare it with two baseline algorithms, viz. the max-loss-rate and the random-rate algorithms. The max-loss-rate algorithm aims to maximize the sum of the losses over all selected agents based on a rate-based beam assignment. Thus, it treats the loss and rate as fixed metrics for vehicle importance. First, it sorts the vehicles in descending order based on their loss $F(W_{G,v})$ and selects as many vehicles as possible until the constraints in (24)–(28) are violated. Each selected vehicle *v* is assigned to the beam $b^{*}$ that it experiences the lowest resource consumption $C_{R,vb^{*}}$. If a vehicle *v* cannot be assigned to its best beam $b^{*}$, the vehicle *v* is not selected for training. The random-rate algorithm is implemented similarly, but the algorithm iterates over the vehicle list in a random order.

One widely used agent selection algorithm from the literature, is the FedCS algorithm, as introduced by Nishio and Yonetani [26], which is based on a greedy method to maximize the number of selected agents. When extending this algorithm to our considered scenario, i.e., vehicle selection and beam allocation, the vehicles need to be assigned to the beam that provides the highest bit rate. Therefore, the FedCS algorithm is almost identical to our proposed VBI-rate algorithm (VBI algorithm when ρ=0). This shows the adaptability of the VBI algorithm in different scenarios via the appropriate configuration of the tuning parameter ρ.

### 5.4. Wireless Scenario

For the wireless communication scenario, we consider an urban macro environment at $f_{C}=3.5$ GHz and a bandwidth of $f_{\mathrm{BW}}=50$ MHz [19,27]. For the wireless propagation in (8), we assume a path loss exponent γ=3.7 and shadowing with σ=8 dB, which are typical values for outdoor dense urban environments [20]. The considered area is covered by M=7 three-sectorized base stations, placed on a hexagonal grid with an inter-site distance of 500 m at a height of 25 m [19]. Each sector is equipped with a 4×4 uniform planar rectangular array (UPRA) configured in a grid of beams comprising $B_{M}=12$ beams.

In urban macro deployments, a high number of vehicles is expected to drive around an urban grid that consists of three blocks, each measuring 433 m by 250 m, making up a total area of 433 m by 750 m [19]. Each street around the block has a total of four lanes and there are two lanes per driving direction. The lane width is 3.5 m [19]. Moreover, the vehicles are driving at a speed of 60 km per hour and their antennas are placed at a height of 1.6 m [27]. At the intersections, the vehicles have a probability of 0.5 to keep driving straight ahead, 0.25 to go left and 0.25 to go right [27]. Finally, each vehicle is equipped with a 2×2 UPRA, which can steer the beam to the direction of the beam formed at the serving sector.

Based on the considered UPRA model, as defined in Appendix A.1, an analysis is conducted in Appendices Appendix A.2 and Appendix A.3 to define the beam directions in the grid of beams at each base station. The derived beam directions are the same at all sectors and they are the following: $-45^{\circ },-15^{\circ },15^{\circ }$ and $45^{\circ }$ in the azimuth plane and $17^{\circ },47^{\circ }$ and $77^{\circ }$ in the elevation plane. A coverage analysis revealed that all roads are covered by the beams pointing at the cell edge. Thus, we only consider the cell edge beams for our evaluation. The remaining UPRA parameters, and in particular the maximum transmit power, noise figure and antenna gain, are derived in Appendix A.3 and summarized in Table 3. These parameters are presented for both the base stations and the vehicles.

Recall from Section 2 that during a given communication round, the vehicles stay connected to one and the same beam. In Appendix A.4, we approximate the time that a vehicle stays connected to a single beam. From the analysis, it is estimated that vehicles stay connected to a cell edge beam between 10.4 s and 17.0 s. This time interval upper bounds the latency budget $\tau_{\mathrm{APP},\mathrm{MAX}}$ to ensure that the assumption of staying connected to one beam is not violated.

In Appendix A.5, we calculate the downlink bit rate at the cell edge at 105 Mbps, which can also serve as a broadcast bit rate. Considering that the FL model size is Z≈107 Mbits the broadcast time duration is $\tau_{\mathrm{DL}}\approx 1.02$ s. Finally, we set the latency budget $\tau_{\mathrm{APP},\mathrm{MAX}}=2.5$ s, which is long enough to allow vehicles to train and upload their local models.

## 6. Results and Discussion

This section evaluates the performance of the VBI algorithm. First, Section 6.1 examines its relative performance compared to the optimal solution of the problem in (29)–(32). Then, we assess the accuracy of the global model when solving the problems in (24)–(28) and (29)–(32). Recall that the accuracy of the global model is defined as the proportion of correctly categorized testing samples divided by the total number of classification instances. For this evaluation, we consider the four learning scenarios described in Section 5.2 and the baseline algorithms presented in Section 5.3. Section 6.2 and Section 6.3 show results for scenarios where vehicles have the same and different data set sizes, respectively. Finally, Section 6.4 compares the four learning scenarios in terms of the time required to reach a certain accuracy level. We present the results of the four learning scenarios as an average of 15 independent simulations. The source code used to generate these results is available in [28].

### 6.1. VBI Algorithm Relative Performance

We evaluate the VBI algorithm with respect to the problem in (29)–(32), i.e., when vehicles have the same training data set size. This comparison demonstrates that the VBI algorithm can provide an accurate approximation to the optimal solution of the problem in (29)–(32). For evaluation, we compare the performance of the VBI algorithm, in terms of the total vehicle importance $Q_{\mathrm{TOT}}$, against the optimal solution given by the CBC solver. Note that the problem in (29)–(32) aims to maximize the total vehicle importance $Q_{\mathrm{TOT}}$ and thus both the VBI algorithm and the CBC solver return this value as part of their solution. Therefore, the ratio of the total vehicle importance $Q_{\mathrm{TOT}}$ as returned by the VBI algorithm divided by the total vehicle importance returned by the CBC solver indicates how similar the two values are. In the case where the ratio equals to one, the two values are equal and the VBI algorithm returns the same value as the CBC solver. We define this ratio as the ‘relative performance’ metric.

For the comparison, three values of the tuning parameter ρ are considered: the two extreme cases of ρ=0 (i.e., VBI-rate) and ρ=1 (i.e., VBI-loss) and the case of ρ=0.8, which leads to a similar value range for the local loss $F(W_{G,v})$ and the resource consumption $C_{R,vb}$. Moreover, the number of vehicles *V* in the network is also varied. Finally, the obtained results are averaged over 1000 independent simulations.

**Figure 4:**
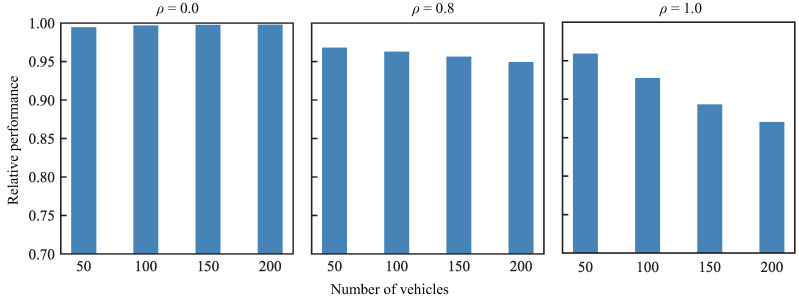
Relative performance of the VBI algorithm to the optimal solution, for the problem where vehicles have the same training data set size.

Figure 4 shows that for the extreme case of ρ=0 (i.e., VBI-rate), the VBI algorithm provides a near-optimal solution, regardless of the number of vehicles in the network. This is because the VBI-rate algorithm selects vehicles with the strongest wireless channels and assigns them to beams with the lowest resource consumption $C_{R,vb}$. Consequently, the number of scheduled vehicles is maximized, leading to a near-optimal total vehicle importance $Q_{\mathrm{TOT}}$.

At the other extreme case of ρ=1 (i.e., VBI-loss), the VBI algorithm prioritizes the selection of vehicles with high loss $F(W_{G,v})$, which might be assigned to a sub-optimal beam, as it was explained in Section 4. Figure 4 shows that the relative performance of the VBI algorithm when ρ=1 is lower than when ρ=0, which is a result of the sub-optimal beam assignment. Specifically, the sub-optimal beam assignment leads to a higher resource consumption which in turn limits the total number of vehicles that can be scheduled and consequently the total vehicle importance $Q_{\mathrm{TOT}}$. Figure 4 also shows that the performance further decreases with the number of vehicles in the network, because there is a higher probability that a vehicle will be assigned to a sub-optimal beam.

When ρ∈(0,1), and specifically ρ=0.8 in this comparison, Figure 4 shows that the relative performance of the VBI algorithm is lower than with ρ=0 but higher than with ρ=1. This is because the tuning parameter ρ configures the vehicle importance $q_{vb}$ to account almost equally for both the resource consumption $C_{R,vb}$ and the loss $F(W_{G,v})$. Therefore, during beam assignment, there is some distinction among the beams to determine the best serving beam in terms of the resource consumption $C_{R,vb}$. However, this distinction is not as prominent as when ρ=0. Hence, the larger the ρ, the less distinction there is among the beams, which consequently leads to a sub-optimal beam assignment. Simulations showed that when ρ=(0,1), the accuracy of the global model falls between the accuracies obtained at ρ=0 and ρ=1. Therefore, in the remainder of this evaluation, we only show the performance of the VBI algorithm when ρ=0 and ρ=1, i.e., the VBI-rate and VBI-loss algorithms, respectively. Therefore, the key takeaway from these evaluations is summarized below:

**Result 1.**  *The VBI algorithm provides a near-optimal solution when ρ→0 (i.e.,* *VBI-rate* *), regardless of the number of vehicles, because it leverages the distinction of beams in terms of resource consumption $C_{R,vb}$. The performance of the VBI algorithm drifts from the optimal solution when increasing the parameter ρ, and in combination with the number of vehicles. Overall, the VBI algorithm offers a good approximation of the optimal solution, and its results will be used in the further evaluations.*

However, the relative performance of the VBI algorithm is not directly related to the accuracy of the global model. Hence, in the following sections, we evaluate the VBI algorithm with respect to global model accuracy.

### 6.2. Same Data Set Size

We evaluate the performance of the VBI algorithm in terms of the accuracy of the global model for the problem in (29)–(32), where the vehicles have the same training data set size. For this purpose, we compare the VBI-rate, VBI-loss, max-loss-rate and random-rate algorithms under scenarios with IID and non-IID data.

#### 6.2.1. IID Data

**Figure 5:**
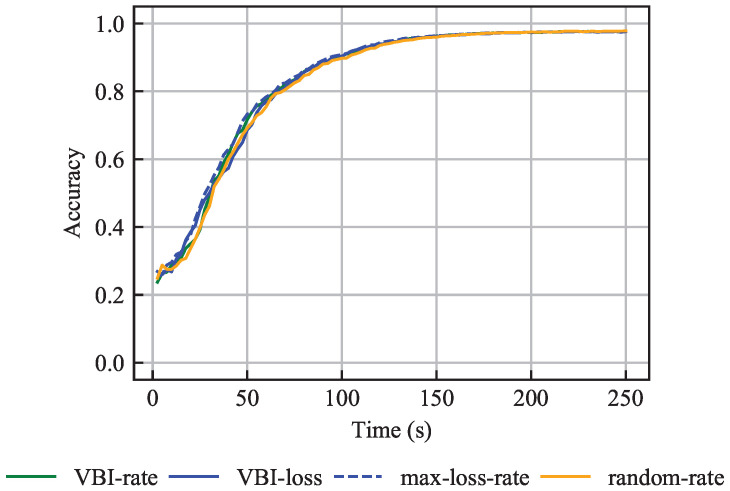
Accuracy over time for the problem with the same training data set sizes and IID data, averaged over 15 independent simulations.

Figure 5 shows accuracy over time and illustrates that all four algorithms achieve similar performance. The comparable results of the VBI-loss and max-loss-rate algorithms are expected, as both select vehicles with the highest loss $F(W_{G,v})$. Additionally, both algorithms select approximately the same number of vehicles per communication round. This implies that the two algorithms are almost identical and that the VBI-loss algorithm mostly assigns, to the selected vehicles, the beam that leads to the lowest resource consumption $C_{R,vb}$. This efficiency arises from the short latency budget $\tau_{\mathrm{APP},\mathrm{MAX}}=2.5$ s, which enforces that only vehicles with favorable wireless channels can participate in the learning process. Due to vehicle mobility, channel quality varies over time, and hence the channel quality limitation due to the latency budget $\tau_{\mathrm{APP},\mathrm{MAX}}$ applies to a different set of vehicles per communication round. Therefore, all four algorithms achieve resource-efficient beam assignments, and all vehicles have fair chances, over time, to be selected.

Moreover, Figure 5 shows that although the VBI-rate and random-max algorithms do not take the loss $F(W_{G,v})$ into account, they perform similarly to the loss-aware VBI-loss and max-loss-rate algorithms. This is because all vehicles have samples from every class, thus making the choice of vehicles less crucial for the learning process. Therefore, the main result herein is the following:

**Result 2.**  *When vehicles have the same data set size and IID data are considered, the choice of vehicles is not crucial, provided that resource-efficient beam assignment is performed.*

#### 6.2.2. Non-IID Data

**Figure 6:**
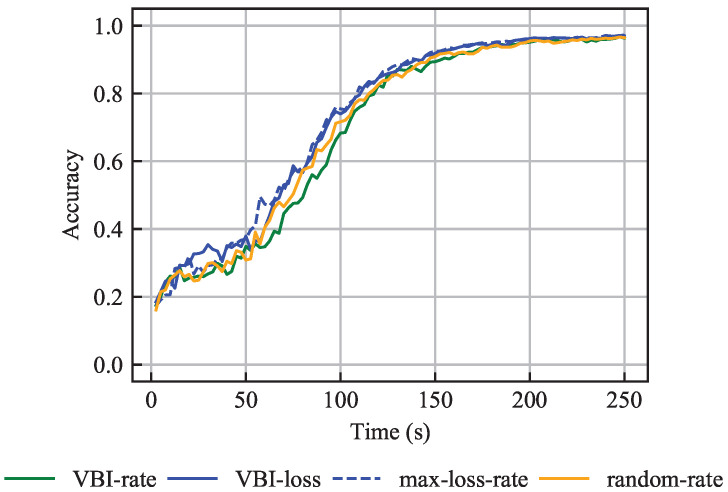
Accuracy over time for the problem with the same training data set sizes and with non-IID data, averaged over 15 independent simulations.

When non-IID data are considered, vehicles contain samples from only two classes; therefore, the selection of vehicles in a given communication round becomes more crucial than in scenarios with IID data. Figure 6 illustrates that, under non-IID data, the loss-aware VBI-loss and max-loss-rate outperform the loss-unaware VBI-rate and random-rate algorithms. This is because the former algorithms take both learning and channel aspects into account. The learning aspect ensures that vehicles carrying samples that contribute more to the learning process are selected more frequently, whereas the channel aspect ensures resource-efficient beam assignment. Recall that the VBI-loss algorithm implicitly takes the channel quality into account via the latency budget $\tau_{\mathrm{APP},\mathrm{MAX}}$. Additionally, Figure 6 shows that the VBI-loss and max-loss-rate algorithms behave almost identically, for the same reason as in the IID data scenario.

Moreover, Figure 6 illustrates that although the four algorithms behave differently, they eventually converge to the same accuracy level. Specifically, an accuracy of 96% is reached within 250 s. Convergence to the same accuracy level occurs because many vehicles are selected per communication round, resulting in sufficient training across all distributed samples. The use of MU-MIMO plays a key role in this outcome by providing two main benefits. First, enhanced throughput allows the FL server to select vehicles that are located at the cell edge. It can be qualitatively argued that these vehicles would not have been selected in a single-antenna system, thus reducing the overall vehicles participation. Second, MU-MIMO enables multiple vehicles per base station to be selected in the same training round, with each vehicle transmitting its model to the base station via a different beam. These benefits allow training on a larger number of samples per communication round, and on a wider sample set. This eventually improves the convergence time of the global model.

Therefore, the key takeaway results are:

**Result 3.**  *When vehicles have the same data set size and non-IID data are considered, loss-aware algorithms provide higher accuracy during the initial learning phase, assuming that resource-efficient beam assignment is performed.*

**Result 4.**  *MU-MIMO-capable base stations improve the convergence time of the global model, as they allow training on a larger number of samples at each communication round, and on a wider sample set. This results from the enhanced quality of the wireless channels, selection of agents at the cell edge, and exploitation of the spatial separation of the vehicles.*

### 6.3. Different Data Set Sizes

We now evaluate the performance of the VBI algorithm in terms of global model accuracy for the problem in (24)–(28), where vehicles contain different training data set sizes. Thus, the vehicles have different training times $\tau_{T,v}$. Although the VBI algorithm does not explicitly select vehicles based on their training time $\tau_{T,v}$, vehicles with shorter training times $\tau_{T,v}$ have a higher selection probability. This is due to constraint (27), enforcing that the selected vehicles need to train and upload their local model within the latency budget $\tau_{\mathrm{APP},\mathrm{MAX}}-\tau_{\mathrm{DL}}\approx 1.5$ s. Therefore, vehicles with short training times $\tau_{T,v}$ can be selected even if their channel quality is not very good. On the other hand, vehicles with long training times $\tau_{T,v}$ can only be selected when they have a very good channel quality.

#### 6.3.1. IID Data

**Figure 7:**
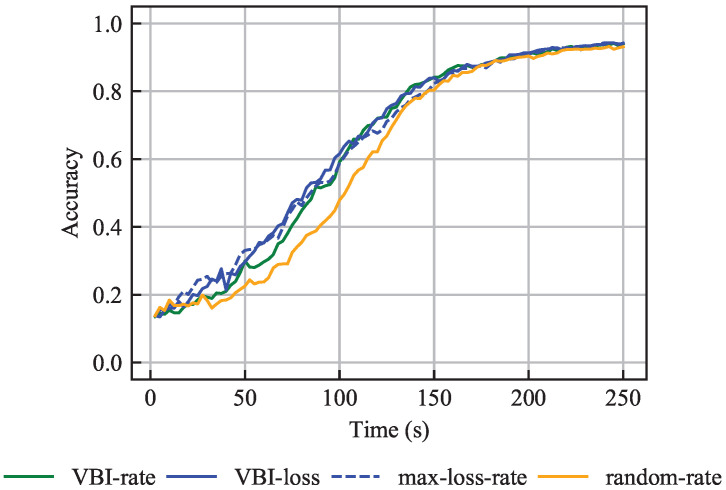
Accuracy over time for the problem with different training data set sizes and with IID data, averaged over 15 independent simulations.

Figure 7 shows the accuracy over time for the four considered algorithms and demonstrates that the VBI-rate, VBI-loss and max-loss-rate algorithms perform similarly, while the random-rate algorithm underperforms. The similarity in performance between the VBI-loss and max-loss-rate algorithms is explained by the applied short latency budget $\tau_{\mathrm{APP},\mathrm{MAX}}$, as noted earlier in Section 6.2.1. Moreover, the design of the two loss-based algorithms and the VBI-rate algorithm allows them to more frequently select vehicles with longer training times $\tau_{T,v}$ compared to the random-rate algorithm. Thus, the random-rate algorithm trains more frequently on a specific set of samples, resulting in a slower convergence compared to the other three algorithms.

Specifically, the VBI-rate algorithm prioritizes vehicles with good channel quality. Therefore, when vehicles with long training times $\tau_{T,v}$ experience good channels, they are likely to be selected. Additionally, the loss-based algorithms prioritize vehicles with higher loss $F(W_{G,v})$. Consequently, vehicles with a high loss $F(W_{G,v})$ are selected for training once their channel quality allows for it. Overall, Figure 7 shows that the three algorithms perform similarly. We conclude that the vehicle selection in scenarios with IID data is not crucial, as long as all vehicles contribute to the learning process, which is consistent with Result 2.

Moreover, vehicles with shorter training times $\tau_{T,v}$ have fewer training samples $K_{v}$. Thus, their contribution to the global model is not very significant. Considering that vehicles with short training times $\tau_{T,v}$ are often selected, the global model does not change significantly per communication round. Therefore, the convergence time is longer compared to the scenario where all vehicles have the same training times $\tau_{T,v}$. The main message in this scenario is:

**Result 5.**  *The good performance of the* *VBI-rate* *,* *VBI-loss* *and* *max-loss-rate* *algorithms, in scenarios where vehicles have different data set sizes and IID data are considered, is attributed to their ability of frequently selecting vehicles with high training times and thus, allow all vehicles to participate in the learning process.*

#### 6.3.2. Non-IID Data

**Figure 8:**
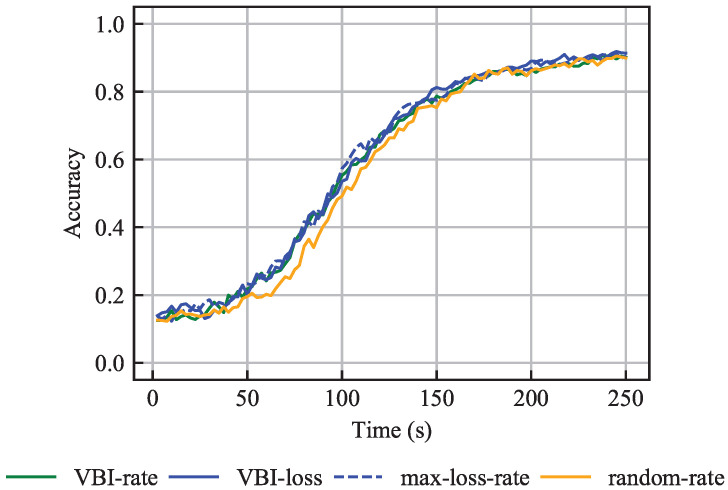
Accuracy over time for the problem with different training data set sizes and with non-IID data, averaged over 15 independent simulations.

Figure 8 shows the accuracy over time for the scenario with non-IID data. Here, the random-rate algorithm again converges slower than the other three algorithms. Similar to the IID case, in Section 6.3.1, the performance difference of the random-rate algorithm compared to the other three algorithms is attributed to not selecting as frequently vehicles with high training time $\tau_{T,v}$. However, this performance difference is more modest in scenarios with non-IID data compared to scenarios with IID data. This occurs because most vehicles are important for the learning process, as their local losses are computed over two classes using a global model averaged across multiple classes. As a result, most vehicles have comparable learning importance, giving the random-rate algorithm a higher likelihood of selecting important ones.

As noted in Result 3, with non-IID data, loss-based VBI-loss and max-loss-rate algorithms provide advantages over VBI-rate and random-rate algorithms. However, when vehicles have different data set sizes, the loss-based algorithms do not provide significant performance gains. The fact that some vehicles have small testing data set size $K_{T,v}$, implies that those vehicles calculate their loss $F(W_{G,v})$ inaccurately. Combined with the non-IID data, where only two classes are represented per vehicle, the loss values in this scenario are both high and highly variable. These two factors indicate that most vehicles are important to participate in the FL training process. As a result, the loss-based algorithms tend to select vehicles more evenly, which reduces the performance gap between them and the VBI-rate and random-rate algorithms, as shown in Figure 8.

Nevertheless, both loss-based algorithms and VBI-rate outperform random-rate, demonstrating that learning importance (loss metric) and wireless importance (rate as wireless resource consumption metric) are equally relevant for FL training on non-IID data with varying training times. Therefore, we conclude that the loss $F(W_{G,v})$ and the rate are similarly important metrics for identifying vehicle importance in the learning process when some vehicles have small testing data set sizes $K_{T,v}$ under non-IID conditions.

To further distinguish among vehicles, one option is to consider their testing dataset sizes $K_{T,v}$. However, this may not yield substantial gains, as it would implicitly prioritize vehicles with long training times $\tau_{T,v}$. Because the uploading time is limited, fewer vehicles would be scheduled. This reduction in the number of scheduled vehicles hinders the learning process and does not allow for faster learning. Thus, we highlight that the loss metric remains a strong indicator of a vehicle’s importance in the learning process, as it selects vehicles with lower training times while achieving the highest accuracy.

The key take away message is:

**Result 6.**  *When vehicles have different data set sizes and non-IID data are considered, the loss $F(W_{G,v})$ remains an effective metric for the learning process, yielding the highest accuracy. However, the loss metric is equally important as the rate metric.*

### 6.4. Comparison of Learning Scenarios

Some applications require training the global model until reaching a specific accuracy target. Therefore, we compare the four algorithms in terms of how much time is needed to reach the 85% and 90% accuracy levels. To average out simulation noise, we consider that the accuracy level is reached if the average accuracy is above the accuracy target for 30 s. Table 4 shows the time in seconds to reach each accuracy level, where a hyphen indicates that the accuracy level could not be reached within the simulated 250 s and have maintained that level for at least 30 s. Specifically, for the scenario addressing problem (24)–(28), where vehicles have different data set sizes and non-IID data, the VBI-loss and max-loss-rate algorithms reach the 90% accuracy target after about 245 s, whereas the VBI-rate and random-rate algorithms have not reached the target yet. However, it can be seen from Figure 8 that all four algorithms reach approximately the same accuracy after 250 s.

Moreover, we conclude from Table 4 that the VBI-loss and max-loss-rate algorithms consistently reach the 90% accuracy target and do so quicker than the other two algorithms. However, the differences between the algorithms are not significant after the initial learning phase, regardless of the learning scenario. This is attributed to the use of MU-MIMO, which improves the quality of the wireless channels and allows the selection of many vehicles per communication round, as also highlighted in Result 4.

Additionally, Table 4 shows that it takes longer to reach the accuracy targets when vehicles have different data set sizes compared to when they have the same data set size. As mentioned in Section 6.3.1, in scenarios with different data set sizes, vehicles with shorter training times are more often selected for training, which then requires more communication rounds to reach a certain accuracy target. Therefore, the comparison among scenarios has the following important take aways:

**Result 7.**  *The two loss-based algorithms, i.e.,* *VBI-loss* *and* *max-loss-rate* *, are stable in terms of reaching the 90% accuracy target more quickly than the* *VBI-rate* *and* *random-rate* *algorithms across all learning scenarios. This is particularly evident with varying data set sizes and non-IID data, where loss-based algorithms show performance similar to* *VBI-rate* *but are more stable.*

**Result 8.**  *In scenarios where vehicles have the same data set size, hence the same training times, the accuracy target is reached faster than when vehicles have different data set sizes, hence different training times.*

## 7. Conclusions

This work investigated the joint vehicle selection and resource allocation problem for FL in vehicular networks with MU-MIMO-capable base stations. Specifically, we described the related optimization problem under two scenarios: when vehicles have the same data set sizes and when they have different data set sizes. To approximate the solution of these optimization problems, we proposed the VBI algorithm. By conducting extensive simulations, we evaluated the VBI algorithm in various learning scenarios and highlighted the key results 1–8. Overall, these results 1–8 showed that loss-based algorithms consistently achieve high accuracy more quickly than the other algorithms, although their performance gains are limited in scenarios where vehicles have different data set sizes and non-IID data. Moreover, we showed that MU-MIMO improves the convergence time of the global model, as highlighted in Result 4.

We further showed that local loss alone does not adequately characterize importance in the learning process under non-IID data and different data set sizes. For future work, it will be of interest to investigate additional learning importance metrics, or combinations of learning and wireless metrics, that may provide further improvements in challenging scenarios with variable data set sizes and non-IID data. In addition, it is worth further investigating which vehicular applications can benefit from federated learning and deriving application-specific latency budgets. Finally, future work should also consider scenarios with energy implications, e.g., optimizing the energy consumption of the network.

## Figures and Tables

**Table 1:** List of most commonly used symbols.

Symbol	Description
$\Gamma_{vb}$	Estimated uplink SNR at vehicle *v* from beam *b* in [dB]
ρ	Constant tuning the relative significance of the learning importance and the resource consumption
$\tau_{\mathrm{APP},\mathrm{MAX}}$	Application-specific latency budget in [s]
$\tau_{\mathrm{DL}}$	Broadcast time of the global model in [s]
$\tau_{T+\mathrm{UL}}$	Time for all selected vehicles to train and upload their local models in [s]
$\tau_{L,b}$	Start time of uplink transmission to beam *b* in [s]
$\tau_{T,v}$	Training time of vehicle *v* in [s]
$\tau_{\mathrm{UL},vb}$	Upload time of vehicle *v* on beam *b* in [s]
$\tau_{B}\in R^{B_{\mathrm{TOT}}}$	Upload latency budget at each beam in [s]
$\tau_{L}\in R^{B_{\mathrm{TOT}}}$	Start time of uplink transmissions on each beam in [s]
$\tau_{T}\in R^{V}$	Training times of vehicles in [s]
$A\in R^{V\times B_{\mathrm{TOT}}}$	Optimization matrix with beam associations between the vehicles and the base station beams
$Q\in R^{V\times B_{\mathrm{TOT}}}$	Importance of vehicles at each beam
$T_{\mathrm{UL}}\in R^{V\times B_{\mathrm{TOT}}}$	Upload times of vehicles at each beam in [s]
$W_{G}$	The weights of the global model
$W_{v}$	The weights of the local model at vehicle *v*
$s\in {\left\{0,1\right\}}^{V}$	Optimization vector for vehicle selection
$B_{M}=\left|B_{m}\right|$	Number of beams at each base station *m*
$B_{\mathrm{TOT}}=\left|B_{\mathrm{TOT}}\right|$	Total number of base station beams in the network
$C_{R,\mathrm{MAX}}$	Available transmission resources
$C_{R,vb}$	Consumption of transmission resources of vehicle *v* on beam *b*
F(·)	The loss function of the model
K=|K|	The total number of samples
$K_{v}=\left|K_{v}\right|$	Number of training samples at vehicle *v*
$K_{T,v}=\left|K_{T,v}\right|$	Number of testing samples at vehicle *v*
M=|M|	Number of base stations in the network
$P_{\mathrm{NF},M}$	Noise figure at the base stations in [dB]
$P_{V,\mathrm{MAX}}$	Maximum transmit power of vehicles in [dBm]
$Q_{\mathrm{TOT}}$	Total vehicle importance
$R_{vb}$	Bit rate at vehicle *v* on beam *b* in [Mbps]
V=|V|	Number of vehicles in the network
$V_{G}=\left|V_{G}\right|$	Number of selected vehicles for training
*Z*	Size of the FL model in [Mbits]
$f_{\mathrm{BW}}$	System bandwidth in [MHz]
$q_{vb}$	Importance of vehicle *v* on beam *b*

**Table 2:** Configuration of the four learning scenarios.

Parameter	Same Data Set Size		Different Data Set Sizes	
	IID	Non-IID	IID	Non-IID
Number of classes per vehicle	10	2	10	2
Training samples $K_{v}$ per vehicle	150	150	150 (average)	150 (average)
Training samples per class per vehicle	15	75 (average)	15 (average)	75 (average)
Testing samples $K_{T,v}$ per vehicle	50	50	50 (average)	50 (average)
Testing samples per class at FL server	100	100	100	100

**Table 3:** Parameters of the UPRA at the base stations and the vehicles.

Base Station		Vehicle	
Parameter	Value	Parameter	Value
$P_{M,\mathrm{MAX}}$	49 dBm	$P_{V,\mathrm{MAX}}$	23 dBm
$P_{\mathrm{NF},M}$	5 dB	$P_{\mathrm{NF},V}$	9 dB
$G_{M,\mathrm{MAX}}$	12 dBi	$G_{V,\mathrm{MAX}}$	6 dBi

**Table 4:** Time, in seconds, needed to reach the 85% and 90% accuracy levels for every algorithm in each learning scenario, where the shortest time per level and scenario is marked with bold.

Algorithm	Same Data Set Size and IID Data		Same Data Set Size and Non-IID Data		Different Data Set Sizes and IID Data		Different Data Set Sizes and Non-IID Data	
	85%	90%	85%	90%	85%	90%	85%	90%
VBI-rate	95	115	145	170	**170**	208	198	-
VBI-loss	95	112	**137**	157	172	**206**	**190**	**243**
max-loss-rate	**92**	**110**	**137**	**155**	175	208	193	248
random-rate	97	117	145	162	180	212	195	-

**Figure A1:**
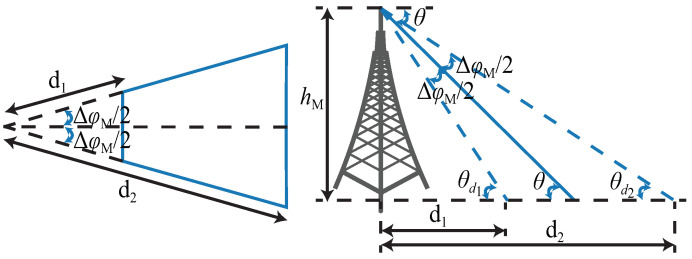
The trapezoid ground area served by a beam with a downwards elevation angle θ and with half-power beamwidth $\Delta \varphi_{M}$, is given by the distances $d_{1}$ and $d_{2}$.

**Figure A2:**
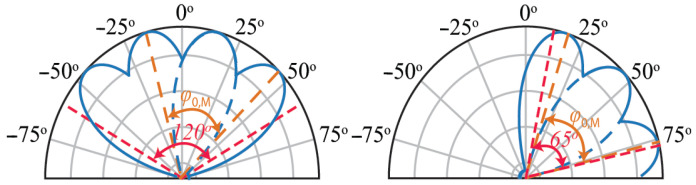
Direction of beams in the GoB for an 4×4 UPRA, in the (**left**) azimuth and (**right**) elevation planes.

**Figure A3:**
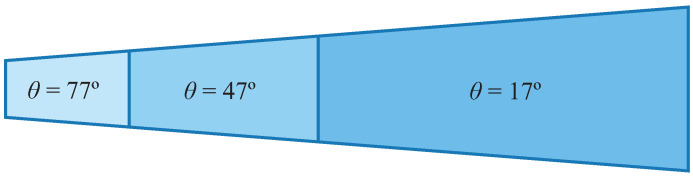
Ground coverage area for every beam in the elevation plane (not in scale), at a given azimuth direction.

## Data Availability

Data can be re-produced by the published code in [28].

## Abbreviations

The following abbreviations are used in this manuscript:

3GPP	Third-Generation Partnership Project
CBC	COIN-OR Branch and Cut
CNN	Convolutional Neural Network
CPU	Central Processing Unit
FL	Federated Learning
FLOP	Floating Point OPeration
GoB	Grid of Beams
IID	Independent and Identically Distributed
ML	Machine Learning
MU-MIMO	Multi-User Multiple Input Multiple Output
OFDMA	Orthogonal Frequency Division Multiple Access
ReLU	Rectified Linear Unit
SGD	Stochastic Gradient Descent
SNR	Signal-to-Noise Ratio
UPRA	Uniform Planar Rectangular Array
VBI	Vehicle Beam Iterative
VGG	Visual Geometry Group

## Appendix A

### Appendix A.1. Antenna Model

In this work, we assume for both the base stations and the vehicles, uniform planar rectangular array (UPRAs) with a total of $N_{E,M}$ and $N_{E,V}$ antenna elements, for the base station antenna arrays and the vehicle antenna arrays, respectively. The antenna elements are positioned at $d_{E}=0.5\lambda$ spacing in both the horizontal and vertical plane, where λ is the wavelength. We assume that each antenna element has an omnidirectional radiation pattern with a gain of $G_{AE}=0$ dBi per antenna element, and the number of antenna elements in the horizontal and vertical plane are equal.

The antenna array at each base station is configured in the grid of beams (GoB) mode. We approximate the beams formed by the vehicles and all beams in the GoB are assumed to have the same half-power beamwidth. Moreover, all beams in the GoB, regardless of their direction, are approximated to have one continuous uniform side lobe. Additionally, given the symmetry of the antenna arrays, the half-power beamwidth $\Delta \varphi_{k}$ and the first-null beamwidth $\varphi_{0,k}$ are assumed to be equal in both the azimuth and elevation planes and they are defined as [29,30]

(A1) $$\Delta \varphi_{k}\approx 2\left[\frac{\pi }{2}-\cos^{-1}\left(\frac{1.391\lambda }{\pi d_{E}\sqrt{N_{E,k}}}\right)\right]\approx \sqrt{\frac{3}{N_{E,k}}},$$

(A2) $$\varphi_{0,k}=2\left[\frac{\pi }{2}-\cos^{-1}\left(\frac{\lambda }{d_{E}\sqrt{N_{E,k}}}\right)\right].$$

The number of beams in the GoB is given by $B_{M}=B_{A,M}B_{E,M}$, where $B_{A,M}$ and $B_{E,M}$ denote the number of beams in the azimuth and elevation planes, respectively, and the number of beams in the GoB is derived from the first-null beamwidth $\varphi_{0,M}$ and the targeted angular range in the azimuth and elevation planes. Specifically, setting the boresight direction of each beam at the null of its adjacent beam, we obtain an angular resolution of $\phi_{B,M}=\frac{\varphi_{0,M}}{2}$, for both the azimuth and elevation planes. Thus, the number of beams in a given plane, i.e., $B_{A,M}$ and $B_{E,M}$, is given by dividing the angular range of the antenna (e.g., for three-sectorized antennas, the angular range in the azimuth plane is $120^{\circ }$) by the angular resolution $\phi_{B,M}$.

The effective beam gains $G_{A,vb}$ and $G_{E,vb}$ experienced by vehicle *v* from beam *b* in the azimuth and elevation planes, respectively, are given by [31]

(A3) $$\begin{array}{cc} G_{A,vb} & =-\min\left\{12{\left(\frac{\varphi_{vb}}{\Delta \varphi_{M}}\right)}^{2},{\mathrm{FBR}}_{M}\right\}+G_{M,\mathrm{MAX}},\\ G_{E,vb} & =\max\left\{-12{\left(\frac{\vartheta_{vb}}{\Delta \varphi_{M}}\right)}^{2},{\mathrm{SLL}}_{M}\right\}, \end{array}$$

where $\varphi_{vb}$ is the angle off the boresight direction of beam *b*, $\vartheta_{vb}$ is the negative elevation angle relative to the direction of beam *b*, ${\mathrm{FBR}}_{M}$ is the front back ratio in dB, $G_{M,\mathrm{MAX}}$ is the maximum gain in dBi and ${\mathrm{SLL}}_{M}$ is the side lobe level in dB, relative to the maximum gain of the main lobe. The maximum beam gain $G_{M,\mathrm{MAX}}$ is given, in dBi, by

(A4) $$G_{M,\mathrm{MAX}}=10\log\left(N_{E,M}\right)+G_{E}.$$

The side lobe gain $G_{S,M}$ of each beam for the UPRA is given, in dBi, by [29]

(A5) $$G_{S,M}=10\log\left(\frac{\sqrt{N_{E,M}}-\frac{\sqrt{3}}{2\pi }N_{E,M}\sin\left(\frac{\sqrt{3}}{2\sqrt{N_{E,M}}}\right)}{\sqrt{N_{E,M}}-\frac{\sqrt{3}}{2\pi }\sin\left(\frac{\sqrt{3}}{2\sqrt{N_{E,M}}}\right)}\right).$$

Therefore, the side lobe level is given, in dBi, by

(A6) $${\mathrm{SLL}}_{M}=G_{M,\mathrm{MAX}}-G_{S,M}.$$

We further assume [29] that ${\mathrm{SLL}}_{M}=-{\mathrm{FBR}}_{M}$. The total beamforming gain at vehicle *v* from beam *b* is then given, in dB, by

(A7) $$\begin{array}{cc} G_{M,vb} & =G_{A,vb}+G_{E,vb}. \end{array}$$

For the transmission beams formed at the vehicles, we assume that they can be steered to the direction of the serving base station beam *b*. Therefore, the vehicle antenna gain $G_{V,vb}$ is equal to the maximum vehicle beam gain $G_{V,\mathrm{MAX}}$, given in dBi by

(A8) $$G_{V,vb}=G_{V,\mathrm{MAX}}=10\log\left(N_{E,V}\right)+G_{E}.$$

### Appendix A.2. Cell Edge Beam Downtilt

This section derives the required beam downtilt to ensure coverage at the cell edge. Based on the angle of the beam on the azimuth and elevation planes, a certain area on the ground can be served. A simplified model to calculate the area on the ground which is served by a beam is to assume a trapezoid ground area, whose size depends on the half-power beamwidth $\Delta \varphi_{M}$ and the downwards elevation angle θ of the beam [32]. Figure A1 illustrates on the left hand side the azimuth projection of the trapezoid ground area, traced with a blue color. The right-hand side of Figure A1 shows with a solid blue line the direction θ of a given beam and the distances $d_{1}$ and $d_{2}$ defining the size of the trapezoid. Specifically, distance $d_{1}$ denotes the distance from the base station until the small base of the trapezoid, and distance $d_{2}$ denotes the distance from the base station until the big base of the trapezoid. Both the distances $d_{1}$ and $d_{2}$ are a function of the beam direction θ, which is constrained as $\frac{\Delta \varphi_{M}}{2}\leq \theta \leq 90^{\circ }-\frac{\Delta \varphi_{M}}{2}$. Additionally, the angles $\theta_{d_{1}}$ and $\theta_{d_{2}}$, which define the distances $d_{1}$ and $d_{2}$, are equal to $\theta_{d_{1}}=\theta +\frac{\Delta \varphi_{M}}{2}$ and $\theta_{d_{2}}=\theta -\frac{\Delta \varphi_{M}}{2}$. Then, the distances $d_{1}$ and $d_{2}$, for the range $\frac{\Delta \varphi_{M}}{2}\leq \theta \leq 90^{\circ }-\frac{\Delta \varphi_{M}}{2}$, are given in meters by

(A9) $$d_{1}=\frac{h_{M}}{\tan\left(\theta +\frac{\Delta \varphi_{M}}{2}\right)},$$

(A10) $$d_{2}=\frac{h_{M}}{\tan\left(\theta -\frac{\Delta \varphi_{M}}{2}\right)}.$$

The cell edge is served by the beam with the smallest downtilt in the elevation plane. Using the method of the trapezoid ground area, we approximate the downtilt, denoted as $\theta_{\mathrm{CE}}$, of the beam serving the cell edge. Using a hexagonal layout, the distance $d_{c}$ from the base station until the cell edge is given by

(A11) $$d_{c}=\frac{2}{3}\mathrm{ISD},$$

where ISD is the inter-site distance. Setting $d_{2}=d_{c}$ in (A10), the downtilt $\theta_{\mathrm{CE}}$ is given by

(A12) $$\theta_{\mathrm{CE}}=\arctan\left(\frac{h_{M}}{d_{c}}\right)+\frac{\Delta \varphi_{M}}{2}.$$

### Appendix A.3. Antenna Array Configuration

We consider 4×4 UPRAs for the base stations and thus $N_{E,M}=16$. Then, the half-power beamwidth $\Delta \varphi_{M}\approx 25^{\circ }$ and the first-null beamwidth $\varphi_{0,M}=60^{\circ }$, based on (A1) and (A2), respectively. Therefore, the angular resolution $\phi_{B,M}=\varphi_{0,M}/2=30^{\circ }$ in both the azimuth and elevation planes.

Considering three-sectorized cells, the antenna arrays need to cover a range of $120^{\circ }$ in the azimuth plane. Consequently, there are $B_{A,M}=120^{\circ }/30^{\circ }=4$ beams in the azimuth plane. On the left hand side of Figure A2, the four beams covering the azimuth plane are shown, pointing at angles $-45^{\circ }$, $-15^{\circ }$, $15^{\circ }$ and $45^{\circ }$.

In the elevation plane, the antenna array needs to cover the vehicles on the ground and thus cover a range of θ less than $90^{\circ }$. Because each base station needs to cover a specific area on the ground, the beams need to point close to the cell edge to ensure that they do not introduce significant interference to the adjacent cells. Based on an ISD = 500 m and antenna height $h_{M}=25$ m, the angle to the cell edge is equal to $4.3^{\circ }$. Therefore, the beams in the elevation plane need to cover a range of $85.7^{\circ }$. Consequently, there are $B_{E,M}=\left\lceil 85.7^{\circ }/30^{\circ }\right\rceil =3$ beams in the coverage range. The beam direction for the cell edge is given by (A12) and thus, the cell edge beam is at $\theta_{\mathrm{CE}}\approx 17^{\circ }$. Based on the angular resolution $\phi_{B,M}=30^{\circ }$, the other two beam directions are $47^{\circ }$ and $77^{\circ }$. The right hand side of Figure A2 illustrates the three beams and their direction.

Based on the derived beam directions, the UPRA at the base stations can simultaneously form and transmit $B_{M}=B_{A,M}B_{E,M}=12$ beams. Furthermore, using (A4), the maximum beam gain $G_{M,\mathrm{MAX}}=12$ dBi and using (A5) and (A6), we calculate ${\mathrm{FBR}}_{M}={\mathrm{SLL}}_{M}=19.1$ dB.

Regarding the vehicles, they are equipped with a 2×2 UPRA and thus $N_{E,V}=4$. Thus, the half-power beamwidth $\Delta \varphi_{V}\approx 50^{\circ }$, as given by (A1). Finally, using (A8), the maximum beam gain $G_{V,\mathrm{MAX}}=6$ dBi.

### Appendix A.4. Beam Connection Time

A vehicle stays connected to the same beam when moving in the area covered by the given beam. The area served by a beam can be calculated using (A9) and (A10) when the half-power beamwidth $\Delta \varphi_{M}$ is substituted by the angular resolution $\phi_{B,M}$. Figure A3 shows the ground coverage area of each of the three elevation beams calculated in Appendix A.3 for a given azimuth direction. Each beam coverage area is a trapezoid and it is described by the distances $d_{1}$ and $d_{2}$, as defined in Appendix A.2. Hence, the two bases and the height of the trapezoid can be calculated. Table A1 shows the length of the distances $d_{1}$ and $d_{2}$ as well as the length of the long base and the height of the trapezoid, considering the angular resolution $\phi_{B,M}=30^{\circ }$ (The distance $d_{2}$ for the beam pointing to the cell edge was set equal to the cell edge distance $d_{c}$, as given in (A11).).

**Table A1:** Geometry description of the trapezoid coverage areas and beam connection time derivation, for the given beam directions.

Beam Direction θ	Distance $d_{1}$ [m]	Distance $d_{2}$ [m]	Long Base [m]	Height [m]	Maximum Connection Time [s]
$16.8^{\circ }$	40.3	333.3	172.5	283.0	10.4–17.0
$46.8^{\circ }$	13.4	40.3	20.9	26.0	1.3–1.6
$76.8^{\circ }$	0.0	13.4	6.9	12.9	0.4–0.8

Table A1 also shows a rough approximation of up to how much time a vehicle will stay connected to the same beam, which depends on the elevation angle of the beam. The maximum connection time interval is calculated as the length of the long base and height of the trapezoid divided by the speed of the car, which is assumed to be 60 km/h.

### Appendix A.5. Broadcast Bit Rate

The broadcast bit rate is chosen such that the associated SNR requirement can be met. In this work, we associate the broadcast bit rate to the cell edge bit rate and hence to the cell edge SNR. We define the cell edge SNR as

(A13) $$\Gamma_{\mathrm{CE}}=P_{B}+G_{T}+G_{V,\mathrm{MAX}}+G_{M}-P_{\mathrm{NOISE}}-P_{\mathrm{NF},V},$$

where $P_{B}$ denotes the broadcast beam transmit power and $P_{\mathrm{NF},V}=9$ dB is the noise figure at the vehicles [27]. Assuming that all four beams at each cell are used for the broadcast and that the maximum transmit power $P_{M,\mathrm{MAX}}=49$ dBm is equally shared among the four beams, we calculate the power $P_{B}=43$ dBm. The distance to the cell edge is $d_{c}=333$ m and thus the path gain $G_{T}=-137.8$ dB. Moreover, from Appendix A.3, the maximum beam gain $G_{V,\mathrm{MAX}}=6$ dBi. For the transmit antenna gain $G_{M}$, we assume the worst case scenario in which the angle off the boresight direction of the beam is equal to $\varphi =\frac{\Delta \varphi_{M}}{2}$ and $\vartheta =\frac{\varphi_{0,M}}{2}$ in the elevation and azimuth planes, respectively. Using (A7), the transmit antenna gain $G_{M}=6$ dB. The noise power is given by

(A14) $$P_{\mathrm{NOISE}}=-174+10\log_{10}\left(B\right),$$

and for a bandwidth of $f_{\mathrm{BW}}=50$ MHz, the noise power $P_{\mathrm{NOISE}}=-97$ dBm. Then, using (A13), the cell edge SNR $\Gamma_{\mathrm{CE}}=5.2$ dB. Therefore, the broadcast bit rate is calculated using (6) as 105 Mbps.
